# Clinical outcome after particle therapy for meningiomas of the skull base: toxicity and local control in patients treated with active rasterscanning

**DOI:** 10.1186/s13014-018-1002-5

**Published:** 2018-03-27

**Authors:** Rami A. El Shafie, Maja Czech, Kerstin A. Kessel, Daniel Habermehl, Dorothea Weber, Stefan Rieken, Nina Bougatf, Oliver Jäkel, Jürgen Debus, Stephanie E. Combs

**Affiliations:** 1 Department of Radiation Oncology, University Hospital of Heidelberg, Im Neuenheimer Feld 400, 69120 Heidelberg, Germany; 2 Deutsches Krebsforschungszentrum (dkfz), Abteilung Medizinphysik, Im Neuenheimer Feld 270, 69120 Heidelberg, Germany; 3 Heidelberg Ion Therapy Center (HIT), Im Neuenheimer Feld 470, 69120 Heidelberg, Germany; 4 Department of Radiation Oncology, Klinikum rechts der Isar, Technische Universität München (TUM), Ismaninger Straße 22, 81675 Munich, Germany; 5 Helmholtz Zentrum München, Department of Radiation Sciences (DRS), Institute of Innovative Radiotherapy (iRT), Ingolstädter Landstraße 1, Munich, Germany; 6 Deutsches Konsortium für Translationale Krebsforschung (DKTK), Partner Site Munich, Munich, Germany; 7 Heidelberg Institute for Radiation Oncology (HIRO), Im Neuenheimer Feld 400, 69120 Heidelberg, Germany; 8 Institute for Medical Biometry and Informatics (IMBI), Heidelberg University Hospital, Im Neuenheimer Feld 130.3, 69120 Heidelberg, Germany; 9 Clinical Cooperation Unit Radiation Oncology (E050), German Cancer Research Center (dkfz), Im Neuenheimer Feld 280, 69120 Heidelberg, Germany

**Keywords:** Proton therapy, Carbon ion therapy, Active raster-scanning, Skull base, Toxicity, Quality of life, Radiotherapy, Radiotolerance, Benign, Malignant

## Abstract

**Background:**

Meningiomas of the skull base account for 25–30% of all meningiomas. Due to the complex structure of the cranial base and its close proximity to critical structures, surgery is often associated with substantial morbidity. Treatment options include observation, aggressive surgical intervention, stereotactic or conventional radiotherapy.

In this analysis we evaluate the outcome of 110 patients with meningiomas of the skull base treated with particle therapy. It was performed within the framework of the “clinical research group heavy ion therapy” and supported by the German Research Council (DFG, KFO 214).

**Methods:**

Between May 2010 and November 2014, 110 Patients with skull base meningioma were treated with particle radiotherapy at the Heidelberg Ion Therapy Center (HIT). Primary localizations included the sphenoid wing (*n* = 42), petroclival region (*n* = 23), cavernous sinus (*n* = 4), sella (*n* = 10) and olfactory nerve (*n* = 4). Sixty meningiomas were benign (WHO °I); whereas 8 were high-risk (WHO °II (*n* = 7) and °III (*n* = 1)). In 42 cases histology was not examined, since no surgery was performed.

Proton (*n* = 104) or carbon ion (*n* = 6) radiotherapy was applied at Heidelberg Ion Therapy Center (HIT) using raster-scanning technique for active beam delivery. Fifty one patients (46.4%) received radiotherapy due to tumor progression, 17 (15.5%) after surgical resection and 42 (38.2%) as primary treatment.

**Results:**

Median follow-up in this analysis was 46,8 months (95% CI 39,9–53,7; Q1-Q3 34,3–61,7). Particle radiotherapy could be performed safely without toxicity-related interruptions. No grade IV or V toxicities according to CTCAE v4.0 were observed. Particle RT offered excellent overall local control rates with 100% progression-free survival (PFS) after 36 months and 96.6% after 60 months. Median PFS was not reached due to the small number of events. Histology significantly impacted PFS with superior PFS after 5 years for low-risk tumors (96.6% vs. 75.0%, *p* = 0,02). Overall survival was 96.2% after 60 months and 92.0% after 72 months from therapy. Of six documented deaths, five were definitely not and the sixth probably not meningioma-related.

**Conclusion:**

Particle radiotherapy is an excellent treatment option for patients with meningiomas of the skull base and can lead to long-term tumor control with minimal side effects. Other prospective studies with longer follow-up will be necessary to further confirm the role of particle radiotherapy in skull base meningioma.

## Background

Meningiomas account for approximately one third of all primary brain tumors and tumors of the central nervous system [1]. Most are benign, slow growing lesions originating from the arachnoidal cap cells, with the skull base being the most frequent localization [2]. Besides benign histology, a smaller number of meningiomas can be of atypical or anaplastic histology, characterized by aggressive growth patterns and a high rate of recurrence [3]. Many analyses focus on meningiomas of the skull base because of its intricate anatomy and the close proximity to vascular structures, cranial nerves and the brainstem; consequently treatment in those cases is challenging and treatment options are controversially discussed.

Surgical resection has for long been the treatment of choice but in the last decades advances in radiotherapy (RT) such as radiosurgery, fractionated stereotactic radiotherapy (FSRT), or intensity-modulated radiotherapy (IMRT) have made radiotherapy an important treatment alternative [4, 5]. Due to the complex anatomy of the skull base, tumor adherence to bony structures and the close proximity to sensitive organs at risk (OAR), total resection is often not possible as it may cause to substantial morbidity. Consequently, as neurosurgical resection is subtotal in many cases, it cannot achieve high long-term local control and overall survival rates [6]. Additional radiotherapy can improve chances for long-term tumor control [7].

Meningiomas are often discovered incidentally or present with only mild symptoms and indolent growth patterns. In those cases, there is no urgent need for therapeutic intervention. Active surveillance can be a reasonable approach, focusing on precise high-resolution contrast-enhanced magnetic resonance imaging (MRI) and clinical examinations in regular intervals (e.g. every six to 12 months, provided an asymptomatic/stable clinical situation). However, if the tumor enlarges significantly during the course of neuroimaging or clinical symptoms develop or worsen, treatment becomes mandatory [6].

Particle beams, such as protons and heavier ion beams like carbon ions offer high precision when it comes to dose application to the tumor volume so that OAR can be very effectively spared [8, 9].

With its unique physical characteristics, including the inverted dose profile, high local dose deposition within the Bragg Peak and a steep falloff outside the treatment volume, particle radiation therapy leads to a greater dose conformity than photon RT [10]. Compared to protons, carbon ions additionally offer the advantage of higher biological efficiency with a relative biological effectiveness (RBE) ranging between 3 and 5, potentially leading to higher local control rates [11].

To date, particle therapy at Heidelberg Ion Therapy Center (HIT) has been integrated into the clinical environment at our institution for close to a decade and is constantly being validated for the treatment of skull base meningiomas. In this present study, we analyse our results for skull base meningiomas in 110 patients treated with particle therapy – protons as well as carbon ions – with special focus on treatment outcome and toxicity.

## Methods

### Patient characteristics and histology

In the present analysis we included 110 consecutive patients with meningiomas of the skull base that had not previously received radiotherapy. All patients received particle therapy – either with proton beams or carbon ion beams – at the Heidelberg Ion Therapy Center (HIT). One hundred four patients received proton therapy, 6 patients received carbon ion radiotherapy. All patients were enrolled in a close follow-up program, consisting of neuroimaging as well as clinical-neurological assessments. According to WHO classification, 60 (54.5%) meningiomas were categorized as benign (WHO grade I). In 8 patients (7.3%) a high-risk histology was observed, including 7 (6.4%) WHO grade II and 1 (0.9%) WHO grade III. In a total of 42 patients (38.2%) histology was unknown, since surgery was not performed. The diagnosis in these cases was based on clinical presentation in combination with imaging, consisting of MRI as well as computed tomography (CT), revealing the typical attributes of meningiomas. In some cases an additional FET or Ga68–DOTATOC-PET examination was performed to support the diagnosis.

### Tumor location

The exact locations of the meningiomas were ascertained by reviewing all images used for treatment planning. Often, tumors had ambiguous extent into structures of the skull base and extended into several regions. In those cases we focused on main tumor extension and tumor origin to create a common classification.Patient characteristics, histology and tumor locations are listed in Table 1.

**Table 1:** Patient characteristics

Patient characteristics	*n*=	Percent
Gender		
male	22	20.0%
female	88	80.0%
Age at radiotherapy		
Mean (SD)	53	11.7
Median (Q1-Q3)	52	29–85
Median (range)	52	45–59
Histology		
WHO I	60	54.5%
WHO II	7	6.4%
WHO III	1	0.9%
unknown	42	38.2%
Location		
sphenoid wing	42	38.2%
petroclival	23	20.9%
sphenoorbital	13	11.8%
supra- and/or parasellar region	10	9.1%
sinus cavernosus	4	3.6%
olfactory	4	3.6%
tentorial fold	3	2.7%
petrosal	5	4.5%
cerebello-pontine angle	3	2.7%
foramen magnum	3	2.7%
Karnofsky performance score		
≥ 80%	101	91.8%
< 80%	9	8.2%
Previous surgery		
partial resection	62	56.4%
no surgery	32	29.1%
unknown	9	8.2%
biopsy	4	3.6%
complete resection	3	2.7%
treatment setting		
due to tumor progression	51	46.4%
definite	42	38.2%
adjuvant / additive	17	15.5%
Particle therapy		
protons	104	94.5%
carbon ions	6	5.5%

### Previous surgery

In most patients, previous neurosurgical intervention had been performed: 69 patients (62.7%) had at least one surgical intervention, which was either subtotal or a biopsy in 66 patients and complete in only 3 patients. Nine patients had an unknown course of surgery. In 29 patients (26.4%) undergoing surgery, more than one intervention had been performed in the past and 8 patients (7.3%) received three or more interventions. In a total of 51 cases (46.4%) RT was performed due to tumor progression, 42 patients (38.2%) received radiation therapy as a definite treatment with some patients undergoing a biopsy beforehand. In 17 patients (15.5%) RT was performed after surgical resection.

### Pre-treatment imaging

For immobilization purposes, an individual custom-made thermoplastic mask was used throughout the entire treatment program. Treatment planning was based on a high-resolution CT scan (native and contrast-enhanced, 3 mm slice thickness). For target volume definition the treatment planning CT imaging data was in all cases matched to a contrast-enhanced MRI with a maximum slice thickness of 3 mm, including a T1-weighted contrast-enhanced sequence to allow for a more precise estimate of tumor extension. In 52 cases (47.3%) an additional FET- and/or DOTATOC-PET was performed to further facilitate target volume definition. Particle irradiation was delivered using active rasterscanning for both protons and carbon ions, applying one fraction a day, 6 days a week.

### Treatment planning for proton therapy

Low-risk meningiomas (WHO grade I or unknown, *n* = 102) were treated with proton therapy. Two patients with higher grade meningeomas (grade II n = 1, grade III n = 1) were also treated with proton therapy. Those patients had previously received radiotherapy for other cranial tumors (retinoblastoma in one case and a different meningioma in a non-overlapping area in the other) and the moderately hypofractionated approach of carbon ion radiotherapy (3 Gy(RBE) per fraction) combined with the higher biological effectiveness was deemed not ideal in those cases with previous dose to OAR. Proton therapy was thus preferred in those two cases. Target volume delineation for proton therapy was performed as follows: On the T1-weighted sequence contrasted tumor formations were delineated as gross tumor volume (GTV). To define the clinical target volume (CTV) a safety margin of 1–2 mm (benign histology) or 5 mm (malignant histology) was added and adapted at the discretion of the treating physician including adjoining meningeal enhancement (dural tail) and areas of potential microscopic spread. An isotropic PTV margin of 3 mm was added in all cases to compensate for positioning and technical insecurities, as is standard procedure for intracranial irradiation at HIT. Details of resulting target volume sizes are illustrated in Table 2. Generally, coverage by the prescribed dose was optimized for CTV; focally reduced PTV coverage was tolerated to allow for OAR sparing in cases of necessity. Median cumulative dose for proton irradiation was 54 Gy(RBE) (range 50–60 Gy(RBE)) at a dose per fraction of 1,8 (*n* = 57) or 2 (*n* = 47) Gy(RBE).

**Table 2:** Target volume sizes

	median (ml)	Q1-Q3 (ml)	mean (ml)	std dev (ml)
Target volumes for proton irradiation				
GTV	22,6	12,5–35,1	28,7	25,9
CTV	31,5	19,2–49,9	44,5	42,4
PTV	50,8	32,7–76,3	68,0	57,5
Target volumes for carbon ion boost irradiation				
GTV	54,6	38,4–59,8	47,3	22,3
CTV	46,9	42–77,8	58,5	29,3
PTV	65,7	59,6–112	82,1	38,8

### Treatment planning for carbon ion therapy

High-risk meningeomas (WHO grades II and III, *n* = 6) were treated with a carbon ion boost after having received a median cumulative dose of 50 Gy (range 48,4–55,8 Gy) of photon irradiation. Target volume delineation and dose prescription were performed analogous to the MARCIE trial, a prospective trial being conducted at our institution for the treatment of atypical meningeomas [3]. For carbon ion irradiation contrast-enhanced areas on the T1-weighted MRI sequence were delineated as GTV with a 5 mm CTV margin that could be adapted at the discretion of the treating physician (e.g. to respect anatomic boundaries). An isotropic PTV margin of 3 mm was added as explained earlier. Details of resulting target volume sizes are illustrated in Table 2. Carbon ion dose prescribed in all cases was 18 Gy(RBE) at a dose per fraction of 3 Gy(RBE).

### Follow-up procedure

Patients were followed up prospectively after completion of particle therapy as described previously [12]. Clinical examinations, including ophthalmological and neurological evaluations if needed and contrast-enhanced MR imaging were scheduled initially 6 weeks after RT. Thereafter, patients were followed up every 3 months in the first year and then every 6–12 months in the following years when no clinical or imaging-based signs of tumor progression occurred. Procedure for every follow-up visit would consist of a contrast-enhanced MRI as well as thorough clinical check-up. To complete or update follow-up information, we contacted patients lost to follow-up directly by phone or correspondence asking for clinical neurological assessments as well as current medical imaging. Four patients could not be contacted and were lost to follow-up.

Symptoms and toxicities were documented in detail in the patient’s medical record and subsequently entered into a prospective research database maintained at our institution for long-term systematic follow-up of radiooncological patients [12]. Symptoms were classified according to the Common Terminology Criteria for Adverse Events (CTCAE) v4.0 [13]. New or worsening symptoms were considered acute and treatment-related toxicities if they occurred within the first 6 months after radiotherapy and late toxicities if they occurred after that. Symptoms were followed up and outcome was judged clinically as either stable/improved or worsened. Toxicities of grades I and II according to CTCAE were classified low-grade. Any de novo symptoms grade III or higher were classified high-grade, as were any pre-existing symptoms worsening by at least two CTCAE grades except if directly attributable to tumor progression.

### Statistics

For descriptive baseline analyses, continous variables are given as means (SD) and median (quartiles, range where appropriate) and categorical variables as absolute and relative frequencies. The median follow-up time was calculated using the reverse Kaplan-Meier method [14]. Overall survival (OS) and progression-free survival (PFS) were both determined using the actuarial method of Kaplan-Meier. OS was calculated from the date of the first diagnosis to last follow-up or death and separately from the beginning of radiotherapy to last follow-up or death. PFS was determined from the day of first RT to the date of occurrence of one of the following: last follow-up/tumor progression/death. Survival curves for prognostic factors were compared using a two-sided log-rank test. Since this was a retrospective exploratory data analysis *p*-values are of descriptive nature. A descriptive p-value of < 0.05 was considered to indicate statistical significance. Toxicity was classified according to CTCAE v4.0 and assessed descriptively, giving absolute and relative frequencies for each symptom. Improvement or worsening of a certain symptom was judged clinically on a case-by-case basis. Statistical analyses were performed with the software *IBM SPSS Statistics Version 22 (New York, USA).*

## Results

### Local tumor control and survival

**Fig. 1:**
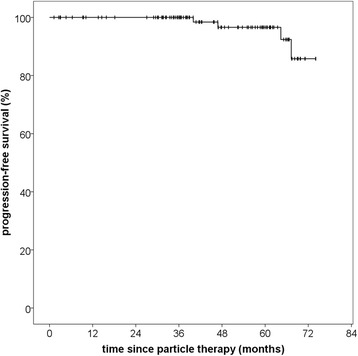
Progression-free survival for patients with skull base meningiomas treated with paticle therapy, regardless of histology

**Fig. 2:**
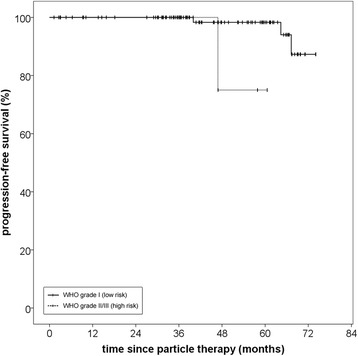
Progression-free survival for patients with skull base meningiomas separated by low risk (WHO grade I) and high-risk (WHO grades II and III) histology

The reverse Kaplan-Meier estimate for median follow-up was 46,8 months (95% CI 39,9–53,7; Q1-Q3 34,3–61,7) for progression-free survival and 57,97 month (95% CI 50,6–62,5; Q1-Q3 38,3–68,9) for overall survival. Progression-free survival rate for all patients treated with particle therapy was 100% after 36 months and 96.6% after 60 months. In total, four patients showed local progression. Median PFS was not reached due to the small number of events. Median time to progression was 55,6 months (Q1-Q3 45,2–65,1; range 40,0–67,3 months) (Fig. 1). Histology significantly impacted PFS with inferior PFS at 60 months (75.0% for high-risk vs. 96.6% for low-risk histology, *p* = 0,02) (Fig. 2), although notably there were only eight patients and one event in the high-risk group, adding to the fact that histology at primary diagnosis was unknown for 38.2% of the patients and thus limiting the conclusions to be drawn on this data regarding the impact of histology.

**Fig. 3:**
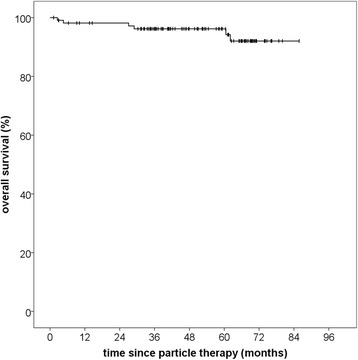
Overall survival calculated from beginning of particle therapy for patients with skull base meningiomas treated with particle therapy, regardless of histology

**Fig. 4:**
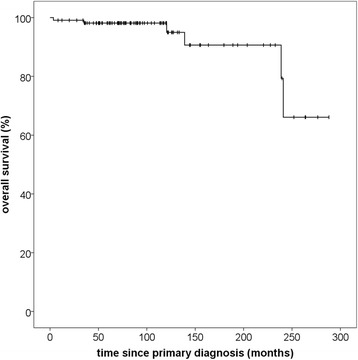
Overall survival calculated from primary diagnosis for patients with skull base meningiomas treated with particle therapy, regardless of histology

Overall survival from the beginning of particle therapy was 96.2% after 60 months and 92.0% after 72 months. Median OS was not reached due the small number of events (Fig. 3). OS from the date of initial diagnosis was 98.1% after 10 years and 90.7% after 15 years (Fig. 4). In total, there were six deaths, for five of which the cause could reliably be determined. None of those deaths was meningioma-related. Two patients died of other oncologic illnesses (pancreatic (*n* = 1) and ovarian cancer (*n* = 1)). Two patients died of substantial cardiovascular co-morbidities and one patient died of a pre-existing advanced-stage normal pressure hydrocephalus. The latter three patients had already been severely reduced in general performance prior to therapy. Four deaths occurred within the first 3 years of radiotherapy. Five deaths occurred in the low-risk group and one in the high-risk group. None of the patients who died had progressed at the last recorded follow-up.

### Progressing patients

The four patients progressing after particle RT were characterized as follows: The first patient was treated for a meningioma of the intrasellar region in a definite setting with 32 × 1,8 Gy(RBE) of proton irradiation without prior surgery or biopsy. She progressed locally 39 months thereafter and underwent partial resection, showing a grade II meningioma, and afterwards received additive re-irradiation with 15 × 3 Gy(RBE) carbon ion therapy. The second patient received 25 × 2 Gy photon IMRT and a carbon ion boost of 18 Gy(RBE) (cumulative dose 68 Gy(RBE)) after multiple partial resections of a petrosal WHO grade II meningioma. Local progression occurred after 47 months and was treated with 15 × 3 Gy(RBE) carbon ions as re-irradiation. He showed no further tumor progression until last follow-up 17 months thereafter. The third patient was treated with 28 × 2 Gy(RBE) of proton irradiation for a meningioma of unknown histology located in the left sphenoid wing and parasellar region. Local progression occurred after 63 months and the patient was referred to neurosurgical resection. The fourth patient received 27 × 2 Gy(RBE) of proton irradiation for a partially resected WHO grade I meningioma of the sphenoorbital region. Local progression occurred after 66 months and the patient was referred to neurosurgical resection.

### Treatment-related toxicity

Treatment was overall well tolerated. All patients completed treatment successfully and no interruptions for toxicity-associated reasons were necessary. No treatment-related grade IV or V toxicities according to CTCAE v4.0 were observed. Acute treatment-related toxicity was mild and mostly resolved within the first 6 months after therapy completion. Among the most common symptoms were focal alopecia (63.6%, *n* = 70), moderate fatigue (47.3%, *n* = 52), focal skin irritation (40.0%, *n* = 44) and headaches (22.7%, *n* = 25). There were two cases of acute grade III toxicity: One case of severe ulcerating mucositis requiring hospitalization and one case of prolonged nausea due to intracranial pressure requiring the administration of corticosteroids. Both cases were controllable by supportive medication. The most common symptoms among late toxicity were fatigue and headaches (both 9.1%, *n* = 10). No more than 10 patients reported any one symptom as late toxicity. Overall, four cases of late CTCAE grade III toxicity were reported: One patient with a parasellar/intrasellar meningeoma developed massively progressive fatigue for which the reason proved to be radiogenic hypopituitarism. Symptoms were controlled after endocrinologic follow-up and adequate medication. Three patients developed radionecrosis, two of them symptomatic (headaches, dizziness). Delivered doses were 30 × 1,8 Gy(RBE) protons (n = 1, interval = 9 months), 27 × 2 Gy(RBE) protons (n = 1, interval = 36 months) and 5 × 3 Gy(RBE) carbon ion boost after 50 Gy photon IMRT (*n* = 1, interval = 7 months). To treat radionecrosis, two patients received high-dose corticosteroids and one patient received Bevacizumab under which clinical and radiologic response could be observed. An overview of acute and late treatment-related toxicity including respective CTCAE gradings is presented in Table 3.

**Table 3:** Acute and late treatment-related toxicity

	Acute treatment-related toxicity				Late treatment-related toxicity			
Side effect	low grade (CTCAE I-II)		high grade (CTCAE III or higher)		low grade (CTCAE I-II)		high grade (CTCAE III or higher)	
	*n=*	*%*	*n=*	*%*	*n=*	*%*	*n=*	*%*
focal alopecia	70	63.6%	0	0.0%	1	0.9%	0	0.0%
fatigue	52	47.3%	0	0.0%	10	9.1%	1	0.9%
skin irritation	44	40.0%	0	0.0%	0	0.0%	0	0.0%
headache	25	22.7%	0	0.0%	10	9.1%	0	0.0%
nausea	23	20.9%	1	0.9%	0	0.0%	0	0.0%
facial pain	12	10.9%	0	0.0%	2	1.8%	0	0.0%
dysgeusia	8	7.3%	0	0.0%	4	3.6%	0	0.0%
lymphedema	7	6.4%	0	0.0%	3	2.7%	0	0.0%
xerostomia	5	4.5%	0	0.0%	4	3.6%	0	0.0%
mucositis	1	0.9%	1	0.9%	1	0.9%	0	0.0%
radionecrosis	0	0.0%	0	0.0%	0	0.0%	3	2.7%

### Symptom response to treatment

The majority of patients presented with multiple symptoms pre-existing radiation therapy which included motoric and sensory impairment, as well as partial trigeminal and facial nerve paralysis, hearing impairment, headache and dizziness. The most common symptom prior to radiotherapy proved to be visual impairment, mostly double vision in 45 patients. Moreover, pre-existing double vision and headache prior to radiotherapy showed the most sizeable improvement, with symptom improvement or stabilization in 34.5% (*n* = 38) for vision impairment and 41.8% (*n* = 46) for headaches. These improvements of pretherapeutic symptoms were achieved within a year after irradiation. No more than 8.2% (*n* = 9) of all patients reported a worsening of any given symptom. The most common among worsening symptoms after RT were headaches (8.2%, *n* = 9) and fatigue (7.3%, *n* = 8). An overview of predominant symptoms prior to particle therapy, their grading according to CTCAE v4.0 and their relative development during follow-up is presented in Table 4.

**Table 4:** Predominant symptoms prior to particle radiotherapy and their relative improvement development during follow-up

	Symptoms before particle irradiation				Symptoms at last follow-up				Clinical outcome			
Predominant clinical symptomps	low grade (CTCAE I-II)		high grade (CTCAE III or higher)		low grade (CTCAE I-II)		high grade (CTCAE III or higher)		stable or improvement		worsening	
	*n*=	%	*n*=	%	*n*=	%	*n*=	%	*n*=	%	*n*=	%
visual impairment	35	31.8%	10	9.1%	28	25.5%	11	10.0%	38	34.5%	6	5.5%
headaches	35	31.8%	0	0.0%	27	24.5%	1	0.9%	46	41.8%	9	8.l2%
hearing impairment	25	22.7%	3	2.7%	25	22.7%	2	1.8%	33	30.0%	3	2.7%
motoric impairment	19	17.3%	1	0.9%	15	13.6%	2	1.8%	20	18.2%	1	0.9%
sensory impairment	14	12.7%	0	0.0%	12	10.9%	0	0.0%	16	14.5%	2	1.8%
dizziness	13	11.8%	0	0.0%	9	8.2%	0	0.0%	21	19.1%	2	1.8%
facial pain	13	11.8%	0	0.0%	9	8.2%	0	0.0%	21	19.1%	2	1.8%
cognitive impairment	11	10.0%	1	0.9%	14	12.7%	2	1.8%	16	14.5%	4	3.6%
seizures	9	8.2%	1	0.9%	2	1.8%	1	0.9%	9	8.2%	1	0.9%
fatigue	6	5.5%	0	0.0%	15	13.6%	1	0.9%	42	38.2%	8	7.3%
nausea	4	3.6%	0	0.0%	9	8,2%	0	0,0%	22	20.0%	0	0.0%
mucositis	0	0.0%	0	0.0%	1	0,9%	1	0,9%	2	1.8%	0	0.0%

## Discussion

The present manuscript evaluates the efficacy and toxicity profile of particle therapy for the treatment of 110 consecutive patients over a a period of 5 years, treated at a single institution. Histology was predominantly benign (WHO grade I) and mainly proton therapy was used, although a combination of photon IMRT and a carbon ion boost was used for a total of six patients with higher-grade histology. None of the treated meningiomas had previously been irradiated. Excellent overall local control at the cost of very light toxicity was achieved with 100% PFS after three and 96.6% PFS after 5 years and histology appearing to significantly influence PFS.

The treatment of skull base meningiomas is a complex clinical situation that requires careful interdisciplinary evaluation. Due to the intricate anatomy of the skull base and the distinct subset of symptoms and toxicities caused by tumors there located, it has been discussed that skull base meningiomas should be regarded as a separate entity regarding outcome and treatment-associated toxicity [4].

Over the years, radiation therapy – and particularly high-precision techniques such as FSRT or IMRT – has evolved to become a central pillar in the multimodal treatment of meningiomas. Several groups have shown high efficacy with minimal toxicity [4, 6, 15]: One of the largest collectives of skull base meningiomas treated with photon IMRT or FSRT and with a median follow-up of 107 months has been described at our institution, showing a local control rate of 95% at 5 years and 88% at 10 years [4]. Histology (WHO grade I vs. grades II and III) proved to be an important prognostic factor, significantly impacting PFS. These data have been confirmed by several similar studies performed at other institutions: Kaul et al. have described PFS to be 93.8% after 5 years for 318 patients with benign meningeomas treated with FSRT [16]. In a separate series focusing exclusively on skull base meningiomas PFS was similar for low-risk histology and 41.8% after 5 years for high-risk histologies [17]. Minniti et al. found a PFS rate of 96% at 3 years and 93% at 5 years in a series of 52 patients with large skull base meningiomas treated with FSRT [18]. Kessel et al. comprehensively reviewed recent literature on the subject and published another large series of 260 patients treated with FSRT or IMRT and including 16% high-risk histologies. They found a PFS rate after 5 years of 87.1% and 54.9% for low-risk and high-risk histologies respectively. Futhermore, patient-reported outcome showed very mild toxicity with no more than 3.0% of patients experiencing worsened or new symptoms ≥3 during RT and the first 6 months thereafter [15].Our results have shown that proton therapy can achieve similarly excellent local control, though continuous long-term follow-up is warranted. Reported data on toxicity and symptom response to treatment is very similar to the results achieved in the current analysis with only mild acute toxicity, the majority of patients showing either stable or improved symptoms during long-term follow-up.

One of the main rationales for the use of particle therapy lies in its higher dose conformity, potentially allowing for better OAR sparing and the reduction of side effects [19–21]. The energy-deposition of accelerated photons occurs continuously over a comparably wide range of penetration depths through tissue [22]. The improved dose distribution of particle therapy is achieved by exploiting the physical characteristics of particle irradiation where the maximum dose deposition occurs within the sharply defined Bragg peak [9]. By varying the particle energy, the position of the Bragg peak can be altered. Particle therapy has been shown to be superior to photon-based techiques in terms of sparing OAR and in terms of target dose homogeneity/conformity with carbon ions showing slightly superior dose distributions compared to protons [23, 24]. Arvold et al. observed a significant dose reduction to neurocognitive, visual and auditory organs achieved by proton irradiation as compared to photon RT. Furthermore, they found protons to reduce the risk of developing a radiologically-induced or associated secondary malignancy by half [1]. Other publications showed a sizeable improvement of pre-exstisting clinical symptoms in up to 47% of patients treated with proton radiotherapy for meningiomas, to which our results compare favorably [10, 25]. We could observe a clear tendency towards improvement that was most prominent in patients suffering from visual impairment, mostly diplopia. 34.5% of all patients showed stabilization or improvement regarding eye-related symptoms and 41.8% regarding headaches, corresponding to 77.8% of the patients reporting pre-therapeutical eye-related symptoms and 94.3% of the patients reporting headaches respectively.

In recent years the body of literature on treating meningiomas with proton therapy has steadily grown and to date there are several publications describing adequate-sized collectives with a median follow-up of 32 to 84 months: Vlachogiannis et al. recently published a retrospective analysis on 170 patients with grade I meningiomas, 155 of which were located at the skull base, who received hypofractionated proton therapy over a period of 13 years. Median follow-up was 84 months and authors reported PFS rates of 93% and 85% at five and 10 years respectively. Main differences in comparison to the current work were the use of passive scattering and the hypofractionated dose regime of 3–8 fractions at 5 or 6 Gy(RBE) per fraction, translating approximately to an EQD2 (equivalent dose in 2-Gy-fractions) of 43 Gy.

Halasz et al. were the first to describe a radiosurgical approach for proton therapy in meningiomas [10]. They analysed a group of 50 patients treated with proton stereotactic radiosurgery at a dose of 13 Gy prescribed to the 90% isodose, achieving a three-year acturial tumor control rate of 94% and toxicity rates similar to those described above. The regarded collective included only small tumor volumes and low-risk histologies. The data suggest that a hypofractionated or even radiosurgical approach, as has been extensively evaluated for photon therapy, might be a feasible and well-tolerated approach for proton therapy as well and achieve satisfactory results [26].

A recent retrospective study by Murray et al. described the outcome of 96 meningioma patients treated with pencil beam scanning proton therapy at Paul Scherrer Institute in Switzerland over a 10 year period [27]. 63.5% were low-risk and and 36.5% high risk meningiomas. The authors reported an estimated 5-year local control (5y-LC) rate of 95.7% for the low-risk group and 68% for the high-risk group, showing consistency with the previously discussed literature and the results of our current work. Five-year grade ≥ 3 toxicity-free survival was 89.1%. The authors reported on identifying several prognostic factors for local failure beside histology (*p* < 0,001), One such factor was the timing of particle therapy (initial vs. for recurrence or progressive disease) with patients treated initially showing favorable outcome; furthermore tumors of the skull base showed favorable outcome vs. non-skull base (*p* = 0,14), as did female patients vs. males (*p* = 0,32). However, none of those factors was tested in multivariate analysis, thus their predictive value should be interpreted with care.

DiBiase and colleagues revealed the size of the GTV to be a significant prognostic factor, since in their described collective of 162 patients treated with Gamma Knife SRS, patients with smaller tumor volumes had longer survival rates with a 5-year overall survival of 100% compared to 59.7% for larger lesions [28].

A small prospective randomized series by Sanford et al. has tested the effect of dose escalation using a combination of photon and proton therapy for the treatment of low-risk meningiomas with a median follow-up of 17,1 years [29]. While overall local control of 98% at 10 years and 90% at 15 years was excellent, no significant benefit could be shown for the use of 63 Gy(RBE) over 55,8 Gy(RBE). However, dose escalation might be beneficial for the treatment of high-risk meningioma patients who show less favorable outcomes with established dose regimens. 5y-LC rate for high-risk meningiomas was 75% in our analysis and though patient number was small, results are comparable to figures reported in recent literature of 50–81% for IMRT or proton therapy, depending on WHO grade [27, 30, 31].

Adeberg et al. could within the high-risk collective identify the WHO grade as prognostic factor for PFS with higher grade yielding inferior PFS (*p* = 0,017) [30]. Notably, the results of McDonald et al. support the rationale of dose escalation for high-risk meningiomas, achieving 5y-LC rates of 87.5% for a radiation dose > 60 Gy(RBE) compared to 50% for ≤60 Gy(RBE) of proton RT (*p* = 0,038) [31]. Regarding dose escalation in a highly radiosensitive region such as the skull-base, the use of heavier ions such as carbon ions with their potentially superior dose distribution and biological advantages attributed to the increased relative biological effiectiveness (RBE) could prove beneficial and may lead to higher local tumor control rates [9, 32]. In a small prospective phase I/II trial conducted at our institution in 2010 on the administration of a carbon ion boost after photon radiotherapy for 10 patients with high-risk meningiomas, we achieved promising results with 5- and 7-year local control rates of 86% and 72% [33]. Median cumulative dose in this analysis was 68 Gy(RBE) and the series included two previously irradiated tumors. Building upon those results, we have initiated the MARCIE trial, a prospective phase II trial evaluating PFS, OS and toxicity for the postoperative bimodal irradiation of atypical meningiomas Simpson grade 4 or 5 [3]. The trial is currently recruiting and the dose regimen of 50 Gy photon RT combined with 6 × 3 Gy carbon ion boost that we applied to the high-risk patients in this analysis is analogous to the concept employed in the MARCIE trial. Though patient number was small for high-risk meningiomas in our current analysis, results are in agreement with previously published data for this dose regimen [33].

Certainly a potential benefit of particle therapy over photon radiation techniques has to be verified clinically and prospective trials are warranted. Several treatment planning studies showed superiority for protons, particularly for larger target volumes: For example, Phillips et al. on reviewing different radiosurgical methods found that particle RT results in supervior dose distributions than photon-based linear accelarator (linac) methods for target volumes > 25 ccm, though for smaller volumes results are comparable while linac methods might offer higher flexibility [34, 35]. Smith et al. supported these findings, comparing linac-based photon RT to Gamma Knife SRS and proton RT and calculating normal tissue complication probability indices (NTCP) based on the dose conformity of the resulting treatment plans and using a logistic model based on the tolerance data by Rubin et al. and Emami et al. [36]. While photon SRS techniques proved superior for small spherical targets, protons had the lowest NTCP for large (> 15 ccm) and peripheral target volumes (13,5 for protons vs. 17,0–33,5 for linac) [37].

To date, our analysis represents the largest group of patients with skull base meningiomas treated with particle therapy, including both protons and carbon ions, in a single institution. Limitations of this analysis include the relatively short follow-up period, its retrospective character and the small number of both high-risk histologies and patients treated with carbon ions, limiting the possibility to perform meaningful subgroup analyses. The median follow-up in this series at 46,8 months – though substantial – is still relatively short when compared to other studies available, especially in the field of precision photon RT. In the light of the benign nature of low-grade meningiomas and predicted long-term tumor-control and overall survival continuous follow-up is warranted. Regarding the different physical and biological characteristics of particle therapy, potential long-term effects are of special interest. Currently particle therapy patients at our institution are included in a close-knit and rigorous follow-up regimen and potential late side effects are documented in a prospective database with dedicated institutional funding for long-term evaluation [12].

To conclusively demonstrate the clinical benefits of particle therapy there is currently a lack of a prospective comparison to advanced photons. Prospective clinical trials have since been initiated at several institutions to further establish the role of particle therapy for the treatment of certain subgroups of intracranial menigiomas.

## Conclusion

In conclusion, particle therapy offers an excellent treatment option for patients with meningiomas of the skull base with long-term tumor control-rates and low toxicity. Compared to outcome reported on the treatment of skull base meningiomas in the literature, the results of our recent study compare favorably. Altough for this entity with its favorable outcome still longer follow-up is warranted, our results are in accordance with previous series of skull base meningiomas treated with particle therapy. Nonetheless, prospective studies with longer follow-up will be necessary to further confirm the role of particle radiotherapy in meningiomas of the skull base. Due to the excellent results with advanced photons an improvement in oncologic outcome by particle therapy in benign meningiomas will be difficult to demonstrate.

## Abbreviations

CTCAE: Common terminology criteria for adverse events
CTV: Clinical target volume
FSRT: Fractionated stereotactic radiotherapy
GTV: Gross tumor volume
IMRT: Intensity-modulated radiotherapy
MRI: Magnetic resonance imaging
OAR: Organ at risk
OS: Overall survival
PFS: Progression-free survival
PTV: Planning target volume
RBE: Relative biological effectiveness
RT: Radiotherapy
SRS: Stereotactic radiosurgery
WHO: World health organization
